# Correlation between* Trichomonas vaginalis* and Concurrency: An Ecological Study

**DOI:** 10.1155/2016/5052802

**Published:** 2016-02-02

**Authors:** Chris R. Kenyon, Deven T. Hamilton

**Affiliations:** ^1^HIV/STI Unit, Institute of Tropical Medicine Antwerp, Nationalestraat 155, 2000 Antwerpen, Belgium; ^2^Department of Medicine, University of Cape Town, Anzio Road, Cape Town, South Africa; ^3^Center for Studies in Demography and Ecology, University of Washington, 206 Raitt Hall, Box 353412, Seattle, WA 98195, USA

## Abstract

*Objective*. There is a large variation in the prevalence of* Trichomonas vaginalis *(TV) between different countries and between racial groups within countries. Sexual partner concurrency may play a role. We investigate the correlation between the prevalence of sexual partner concurrency and TV prevalence.* Methods*. Spearman's correlation to assess relationship between TV prevalence in women and point prevalence of concurrency in men in (1) 11 countries with comparable data (concurrency data from WHO Survey and TV prevalence data from Global Burden of Disease estimates) and (2) three racial groups in the United States (Add Health Study).* Results*. The prevalence of TV and concurrency was positively correlated in the international (rho = 0.84, *P* = 0.001) and USA study (rho = 1.0, *P* < 0.001).* Conclusion*. Prospective longitudinal studies that include measures of partner behavior are required to definitively establish the role of concurrency in the spread of TV.

## 1. Introduction

*Trichomonas vaginalis *(TV) is the most prevalent curable sexually transmitted infection (STI) globally [1]. Its importance lies both in approximately 20% of infections that are symptomatic and in the way that symptomatic and asymptomatic infections are associated with a variety of adverse outcomes including HIV acquisition, preterm labour, and pelvic inflammatory disease [2–5].

Many questions remain around TV epidemiology. Amongst women TV infection has been associated with a number of risk factors such as increasing numbers of partners [2], nonsteady partners [6], current or previous infection with other STIs such as HIV [2], HSV-2 [2], and syphilis [7, 8], sex work, incarceration, and drug use [7]. Some studies have found a positive association [2] and others a negative association with age in women [3, 5, 9]. The incidence of TV is similar in men and women but the prevalence in men is far lower. Thus, the WHO has estimated that just over half the 248 million new TV infections each year occur in men [1]. Men, however, make up only 11% of prevalent TV cases [1]. These discrepancies are explained by biological differences between the sexes (including TVs predilection for oestrogen and menses-related iron [5]) that enable TV to persist for longer and thus result in a higher prevalence in women [5]. Based on various empirical studies STI modelers estimate that the duration of infection in women is 1.12–1.39 years and around 6 weeks in men [3–5, 10–12].

One of the most striking features of TV epidemiology is how strongly it is associated with race. This has been best studied in women in the United States where the adjusted odds ratios (OR) for African American race have been between 5.6 and 13.5 [9, 13–15]. Reviews of the topic have typically postulated multifactorial aetiologies [3, 5, 11]. In this paper we test the hypothesis that sexual partner concurrency, defined by UNAIDS as overlapping sexual partnerships where sexual intercourse with one partner occurs between two acts of intercourse with another partner [16], plays a role in the epidemiology of TV. Novel methodologies have shown the role that concurrency plays in the epidemiology of a number of STIs. These methodologies have included modeling, egocentric sexual network studies, and ecological studies [17–19]. Concurrency facilitates the spread of STIs by reducing the time between acquiring infection and passing it on because one partnership does not have to end before another begins; by removing the protective effect that comes from being earlier in a partner sequence because later partners can now pose an indirect risk to earlier partners when these partnerships overlap in time; and by creating additional paths of infection established by concurrent partnerships [17, 20].

Since sexual networks are population level properties, it is necessary and appropriate to study the correlation between concurrency and STI prevalence at an ecological level [17]. In this paper we assess the correlation between TV prevalence in women and the prevalence of concurrency in men at an international level and between different racial groups within USA.

## 2. Methods

### 2.1. Global Study

#### 2.1.1. Point-Concurrency

Different survey methodologies can yield very different concurrency prevalence estimates [21]. There is only one dataset that we are aware of that used the same methodology to assess the prevalence of concurrency in multiple populations around the world. This is the dataset from the WHO/Global Programme on AIDS (GPA) sexual behavioural survey conducted in 1989/1990. Eleven countries performed these surveys between 1989 and 1990 and asked questions about concurrency. All eleven countries are evaluated here [22, 23]. The surveys all followed WHO/GPA protocols. National probability samples of the general populations aged 15 to 49 were recruited. In two cases, Manila and Rio de Janeiro, the samples were representative of these large cities rather than being nationally representative. All samples were selected based on the probability principle with various designs depending on national factors. A two-stage sampling strategy was used. Census enumeration areas were the first stage and households the second. The sample sizes were 1000–3000 men and women. Response rates were high in all the surveys. The variable for concurrency was derived from the question “Do you now have one or more than one spouse/regular partner?” The concurrency variable we used in our analysis was the percentage of men 15–49 years old who reported having more than one sexual partnership active at the time of the survey. We use concurrency among males rather than females because concurrency has been shown to be a risk factor for transmission; thus, it is concurrency among male partners which we hypothesize is driving higher prevalence of TV among females [24–26].

#### 2.1.2. TV Prevalence

The TV prevalence from the year 1990 was taken from the Global Burden of Diseases Study. For this study the incidence and prevalence of TV were estimated by a literature and database review conducted by the World Health Organization. TV prevalence refers to the percent of women 10 years of age or older who are infected with TV at the beginning of 1990. The figures are age-standardized.

### 2.2. USA Study

Add Health is a longitudinal study of a nationally representative sample of 20,000 adolescents first conducted in 1994. We used data from Wave III, conducted in 2001-2002. Further details of the study are published elsewhere [27]. We used published TV prevalence estimates of women by race. These estimates were based on a urine TV polymerase chain reaction assay [9]. The prevalence of sexually active men reporting two or more partners on the day of the interview by race was calculated using appropriate weighting procedures.

We related the prevalence of TV to the point prevalence of concurrency using Spearman's correlation coefficient. All analyses were conducted with Stata 13.0 (College Station, TX).

## 3. Results and Discussion

**Figure 1:**
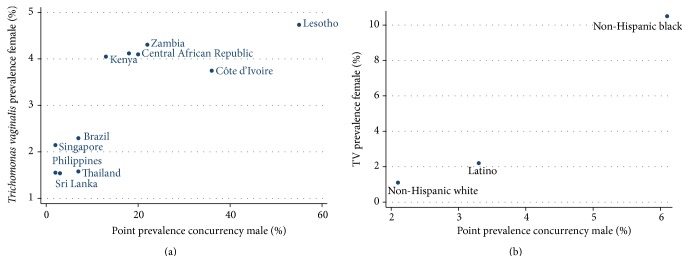
Correlation between the prevalence of* Trichomonas vaginalis *in women and the point prevalence of concurrency in men in (a) 11 countries (rho = 0.84, *P* = 0.001) and (b) racial groups in USA (rho = 1.0, *P* < 0.001)—data sources listed in text.

In the international comparison we found evidence of a positive correlation between the prevalence of concurrency in men and prevalence of TV in women (rho = 0.84, *P* = 0.001, Figure 1). There was also evidence of the same positive correlation in the comparison of different races in USA but this did not meet statistical significance (rho = 1.0, *P* < 0.001, Figure 1).

A positive correlation between concurrency and TV is biologically plausible. Concurrency enhances the transmission of STIs via increasing network connectivity, by removing the protective effect of partner sequencing and accelerating forward transmission [17]. Each of these mechanisms may mitigate the potential natural limiting factor imposed by the short duration of survival of TV in men (6 weeks).

**Figure 2:**
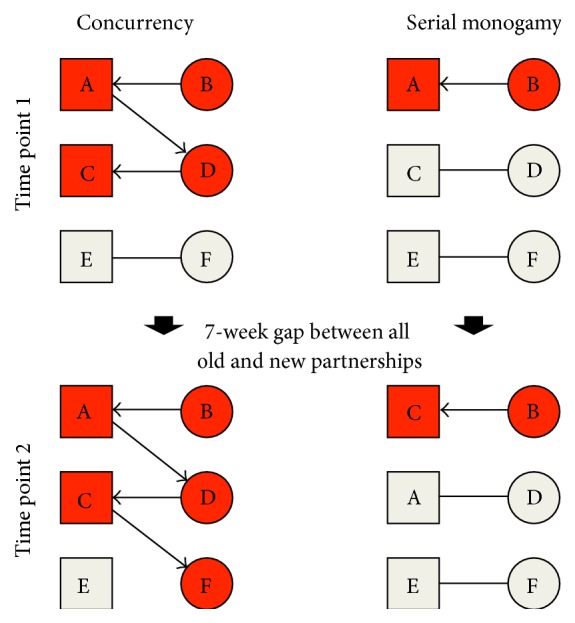
The differential spread of* Trichomonas vaginalis *in two different hypothetical sexual networks following one seed infection in individual B (squares: men, circles: women, red: TV infected, beige: TV uninfected, black bars/arrows: sexual partnership, and direction of arrow: direction of TV transmission; see text for explanation).

This is illustrated in Figure 2, which contrasts TV spread (red) in two different hypothetical sexual networks: high prevalence of long-term concurrent partnerships (“concurrency,” left) and serial monogamy with a 7-week gap between old and new partnerships (“serial monogamy,” right). Individual B is infected at time zero and infects her partner A in both scenarios. In the “concurrency” network, A's concurrency is responsible for transmitting TV to D and then to C. By time point 2 (following a 7-week gap in partnerships) the three women in the “serial monogamy” network now have new partners and only B (a woman) is able to infect one of the new partners. This is partly due to the 7-week gap being longer than the survival time of TV in men. By time point 2 in the “concurrency” network the ongoing connectivity maintains the TV infection in both the males and females. Although the partnership between E and F ended more than 7 weeks previously, F is still able to acquire TV from one of the males (C) due to his ongoing partnership with D. At the end of the scenario, TV prevalence is 5/6 and 2/6 in the “concurrency” and “serial monogamy” networks despite the “serial monogamy” network having had a greater number of partnerships (6 versus 5).

There are a number of limitations to this study. The TV prevalence data from 1990 was based on a literature review conducted by the WHO of TV incidence and prevalence studies conducted using different population samples and diagnostic methodologies. Although the estimation process was adjusted for these, there may have been residual errors. There was however a close correlation between the WHO TV prevalence estimates and those from the only published global review of TV epidemiology that provides country level prevalence estimates [3]. Questions around how many sexual partners one has may be subject to a response bias and it is possible that this could vary between populations. The analysis is purely ecological and thus susceptible to the ecological inference fallacy.

Prospective individual and partner level studies are required to provide a more definitive answer as to what the relationship is between concurrency and TV. Two partnership level studies have reported a link between TV infection and concurrency but both had large limitations. One study found a very high prevalence of self-reported concurrency in the male partners of women diagnosed with TV [28]. Another found a strong association between partner concurrency and a composite STI outcome variable of TV/chlamydia/gonorrhoea [29]. Results were not disaggregated by STI.

TV has been associated with a range of negative health outcomes but the reasons why certain populations are disproportionately affected have not been adequately explained. As such, studies are required to prospectively assess the incidence of TV and associated risk factors including ones pertaining to sexual network structure and position. In the interim it may be worth applying a novel way of analyzing the relationship between STIs and behavior to TV. Hamilton and Morris have recently developed an analytical method that factors in local sex network structure by imputing partner characteristics such as concurrency and assortative mixing [18]. Applying this method to investigate risk factors associated with chlamydia infection in the same Wave III of Add Health that we used, they found that the inclusion of imputed network structure led to a considerable reduction in the large racial differences in chlamydia. It would be interesting to see if similar results were found with TV as the outcome variable.

## 4. Conclusion

We find evidence of a strong ecological level correlation among the prevalence of sexual partner concurrency and* Trichomonas vaginalis.*
